# Organizational management from the perspective of public health: exploring the strategies to improve staff's work enthusiasm and efficiency

**DOI:** 10.3389/fpsyg.2025.1514984

**Published:** 2025-06-19

**Authors:** Zhou Yang

**Affiliations:** Nanjing Agricultural University, Nanjing, China

**Keywords:** psychological state analysis, organizational management, employee work force, efficiency, ways of improvement, public health, overall health

## Abstract

**Background:**

In the evolving landscape of organizational management, employee psychological wellbeing plays a crucial role in driving workforce efficiency and performance. In China, especially within institutions such as universities and public service organizations, this aspect is often underemphasized. This study investigates the relationship between psychological states and employee productivity in the context of Chinese organizational settings.

**Methods:**

We conducted a comprehensive analysis based on psychological assessments, employee interviews, and case studies within selected Chinese institutions. Key psychological indicators such as flow state, anxiety, and stress levels were evaluated, and their influence on employee work force and efficiency was examined.

**Results:**

The analysis reveals a strong correlation between employees' psychological states and their work outcomes. A positive flow state significantly enhances work motivation and creativity, while anxiety and excessive stress hinder efficiency. Targeted strategies including goal-setting, resource provision, environmental support, and psychological tracking mechanisms proved effective in improving overall staff performance.

**Conclusions:**

Integrating psychological state analysis into organizational management practices is essential for fostering a productive workforce. This study provides actionable insights for managers and policy-makers in China, emphasizing the value of mental health support, structured workflow optimization, and cultural environment enhancement to sustainably boost employee motivation and organizational performance.

## 1 Introduction

In the modern era of globalization and technological advancement, organizations face increasing complexity and competition (Gong et al., 2010). Maintaining a motivated and efficient workforce has become essential for long-term success (Dwight et al., 2024). However, traditional management often overlooks employee psychological wellbeing—an aspect now seen as crucial to performance and innovation (Deci and Ryan, 2008). Recent studies emphasize the importance of understanding psychological states in relation to motivation, productivity, and decision-making (Schaufeli and Bakker, 2004). Conditions like flow, anxiety, and stress influence not only individual performance but also organizational adaptability (Liu et al., 2021). While flow enhances focus and creativity, chronic stress and anxiety contribute to burnout and inefficiency (Karasek, 1979).

In China's rapidly evolving institutional and healthcare sectors, these psychological dimensions are increasingly relevant (Lazarus and Folkman, 1987). Organizations face challenges such as high turnover, job dissatisfaction, and mental health issues, particularly in high-pressure fields like education and public service (Fredrickson, 2004). This study explores how psychological state analysis can enhance employee efficiency, with a focus on Chinese organizational settings (Podsakoff et al., 2007). By examining psychological indicators and their management implications, we present a strategic framework to improve wellbeing and competitiveness in line with public health principles (Tuckey and Neall, 2014).

Our study is grounded in Csikszentmihalyi's flow theory, the transactional model of stress and coping (Sun et al., 2023), and the Yerkes–Dodson law (Hobfoll, 2011), which explain how cognitive and emotional states shape work behaviors (Grant and Parker, 2009). These models guide the design of our psychological assessments and intervention strategies (Kahn, 1990). In recent years, China has emphasized workplace mental health in its national agenda (Boyatzis and McKee, 2005), through initiatives like Healthy China 2030 (Bakker et al., 2021) and regulations such as the Mental Health Law of the People's Republic of China (2013), which mandate supportive practices for employee wellbeing.

Despite growing attention to workplace mental health, many studies focus on isolated psychological factors or Western contexts (Sun et al., 2023). There remains a lack of research on integrating flow, anxiety, and stress into strategic HR management in Chinese institutions (Zhang and Li, 2019). This gap is critical given the rising pressure in sectors like education and healthcare and the absence of psychological tracking tools (Csikszentmihalyi, 1990). Our study addresses this by linking multidimensional psychological states with real-time efficiency data (Yerkes and Dodson, 1908). Positioned in a Chinese context, the research offers culturally relevant strategies aligned with national priorities such as Healthy China 2030. It contributes both theoretically and practically to HRM innovation in emerging organizations (Gile et al., 2022b).

In addition to classical constructs like flow and anxiety, newer frameworks highlight motivational and resource-based approaches to performance. den Broeck et al. (2021) expanded Self-Determination Theory (SDT) by identifying autonomy, competence, and relatedness as key to intrinsic motivation. reviewed leadership's role within the Job Demands–Resources (JD-R) Model, showing how high demands lead to burnout unless balanced by managerial support and recognition (Huang et al., 2025). Our study integrates real-time psychological tracking with these theories to analyze how psychological states emerge under different demand–resource conditions (Sun et al., 2025), offering a comprehensive perspective for Chinese organizational contexts.

## 2 Methods

### 2.1 Preliminaries

This study employed a mixed-methods research design to capture both quantitative indicators and qualitative insights regarding employee psychological states and performance. The quantitative component involved structured psychological assessments administered through digital surveys, while the qualitative component was embedded in follow-up interviews and contextual observations related to managerial interventions. Participants were selected using purposive sampling to ensure coverage across key departments—Sales, R&D, and HR—within a mid-sized enterprise in China. The organization granted access and facilitated data collection over a six-month period, from September 2023 to February 2024. Psychological assessments were conducted at three intervals to enable longitudinal tracking, and included standardized Likert-scale items measuring depression, anxiety, stress, job satisfaction, and work-life balance. Qualitative feedback from employees and HR managers was collected through one-on-one sessions to contextualize the survey findings and inform targeted interventions.

The study was conducted within a privately-owned mid-sized enterprise located in Eastern China, operating in the professional services sector. A total of 68 employees participated in the research. Participants were selected using purposive sampling to ensure cross-departmental representation from Sales (*n* = 24), Research and Development (*n* = 21), and Human Resources (*n* = 23). Demographic information was collected to assess the diversity of the sample. Among the participants, 59% were male and 41% female; the average age was 34.2 years (SD = 6.3), with ages ranging from 25 to 48. Regarding education, 64% held a bachelor's degree, 28% had a master's degree, and 8% completed vocational or college-level training. Participants held various job roles including junior staff, mid-level professionals, and department supervisors. Participation was voluntary, and all data were anonymized. The study was reviewed and approved by the company's internal ethics panel prior to data collection.

To ensure conceptual clarity and facilitate replication, we have provided operational definitions for all key terms and variables used in this study. These include constructs such as holistic health of employees, work enthusiasm, organizational management, and strategic resource allocation. A comprehensive overview of these terms, along with their contextual meaning and measurement approaches, is presented in Table 1.

**Table 1:** Operational definitions of key concepts and variables.

**Term**	**Definition**	**Operationalization in this study**
Holistic health of employees	A comprehensive state of wellbeing including psychological, emotional, and professional aspects	Measured through six subdimensions: flow, anxiety, stress, job satisfaction, work-life balance, and professional development needs. Aggregated via the Mental Wellness Index (MWI)
Staffs	Employees within the organization	Refers to full-time professionals from Sales, R&D, and HR departments. Support and part-time staff were excluded
Work enthusiasm	Psychological energy and motivation toward work tasks	Assessed via flow state indicators and qualitative feedback on engagement, focus, and task persistence
Organizational management	Strategic administration of human and structural resources to enhance performance	Refers to the systematic integration of psychological diagnostics (CognityTrack) into HRM and performance allocation processes
Strategic resource allocation	Data-informed distribution of operational support and tasks	Implemented using LPEC metrics and intervention vectors (PsyAlign), including reallocation of roles, training, and counseling

### 2.2 CognityTrack model

In response to the increasing complexity of employee psychological dynamics in organizational contexts, we propose a novel diagnostic framework named CognityTrack, designed to monitor, quantify, and interpret latent psychological signals that shape workforce efficiency and engagement. CognityTrack represents a psychometric-aware, context-sensitive modeling approach that transcends traditional human resource metrics by embedding validated psychological instruments within a feedback-driven organizational architecture.

CognityTrack integrates digital assessment protocols, psychometric modeling, and individualized profiling to create a closed-loop tracking system that feeds directly into managerial decision-making. Unlike traditional frameworks that treat job satisfaction and stress in isolation, this model operates on multidimensional constructs rooted in flow theory, cognitive-affective theory, and job-demands-resources (JD-R) model. The framework systematically links quantitative psychological states with task allocation, role matching, and performance design, thereby enabling real-time optimization of workforce deployment.

Let the psychological state vector of an employee *i* at time *t* be defined as:


(1) $$\begin{array}{l} {\text{p}}_{i}^{\left(t\right)}=\left[\begin{array}{c} f_{i}^{\left(t\right)}\\ a_{i}^{\left(t\right)}\\ s_{i}^{\left(t\right)}\\ j_{i}^{\left(t\right)}\\ w_{i}^{\left(t\right)}\\ d_{i}^{\left(t\right)} \end{array}\right]\in {\mathbb{R}}^{6} \end{array}$$

where *f* denotes flow state score, *a* is anxiety, *s* is stress, *j* is job satisfaction, *w* is work-life balance, and *d* is professional development demand.

Each component is derived from a normalized Likert-scale input, scaled to the interval [0, 1] through min–max transformation:


(2) $$\begin{array}{l} x_{i}^{\left(t\right)}=\frac{x_{i,\text{raw}}^{\left(t\right)}-x_{\text{min}}}{x_{\text{max}}-x_{\text{min}}},\;x\in \left\{f,a,s,j,w,d\right\} \end{array}$$

We define a composite mental wellness index (MWI) for individual *i*:


(3) $$\begin{array}{l} {\text{MWI}}_{i}^{\left(t\right)}=\omega_{1}f_{i}^{\left(t\right)}-\omega_{2}a_{i}^{\left(t\right)}-\omega_{3}s_{i}^{\left(t\right)}+\omega_{4}j_{i}^{\left(t\right)}+\omega_{5}w_{i}^{\left(t\right)} \end{array}$$

subject to:


(4) $$\begin{array}{l} \overset{5}{\underset{k=1}{\sum }}\omega_{k}=1,\;\omega_{k}\in \left[0,1\right] \end{array}$$

This index serves as a central node in evaluating latent psychological efficiency capacity (LPEC):


(5) $$\begin{array}{l} {\text{LPEC}}_{i}^{\left(t\right)}=\phi \left({\text{MWI}}_{i}^{\left(t\right)},\nabla {\text{p}}_{i}^{\left(t\right)}\right) \end{array}$$

where the gradient operator tracks temporal change:


(6) $$\begin{array}{l} \nabla {\text{p}}_{i}^{\left(t\right)}={\text{p}}_{i}^{\left(t\right)}-{\text{p}}_{i}^{\left(t-1\right)} \end{array}$$

To classify psychological profiles, we adopt unsupervised clustering based on Euclidean distance across the state vectors:


(7) $$\begin{array}{l} C=\left\{C_{1}:\text{stable-high},C_{2}:\text{fluctuating-risk},C_{3}:\text{low-engagement},\ldots \right\} \end{array}$$

For role-task fit, we define a role-pressure matrix **R**∈ℝ^*n*×*m*^:


(8) $$\begin{array}{l} R_{ij}=\gamma \cdot \left(1-f_{i}^{\left(t\right)}\right)+\delta \cdot s_{i}^{\left(t\right)}+\zeta \cdot a_{i}^{\left(t\right)}-\eta \cdot j_{i}^{\left(t\right)} \end{array}$$

with corresponding assignment optimization:


(9) $$\begin{array}{l} \underset{\text{A}}{\min}\underset{i,j}{\sum }A_{ij}\cdot R_{ij}\;\text{subject to }\text{A}\in {\left\{0,1\right\}}^{n\times m},\underset{j}{\sum }A_{ij}\leq 1 \end{array}$$

Survey data were collected longitudinally:


(10) $$\begin{array}{l} P=\left\{{\text{p}}_{i}^{\left(t\right)}|i\in \left[1,n\right],t\in \left\{t_{1},t_{2},t_{3}\right\}\right\} \end{array}$$

Finally, personalized intervention logic is specified as:


(11) $$\text{Intervene}(i)=\{\begin{array}{ll} \text{Training}, & \text{if }d_{i}^{\left(t\right)}>\theta_{1}\wedge j_{i}^{\left(t\right)}<\theta_{2}\\ \text{Counseling}, & \text{if }a_{i}^{\left(t\right)}>\theta_{3}\\ \text{Promotion Pathing}, & {\text{if MWI}}_{i}^{\left(t\right)}>\theta_{4}\wedge f_{i}^{\left(t\right)}>\theta_{5} \end{array}$$

CognityTrack thus serves as a theoretical and operational framework for transforming real-time psychological data into actionable workforce strategies. It provides a scalable and quantifiable model for embedding human-centric metrics into the heart of organizational design.

### 2.3 PsyAlign strategy

Building upon the diagnostic outputs of the CognityTrack model, we introduce the PsyAlign Strategy, a novel adaptive intervention architecture designed to align psychological readiness with operational task demands through continuous data-feedback loops. PsyAlign is not a static intervention scheme, but a dynamic mapping strategy that adjusts allocation, training, and leadership responses based on longitudinal psychometric data. It represents a translational layer from measurement to action, grounded in behavioral optimization theory and organizational cybernetics.

Let each employee *i* at time *t* be associated with a psychological efficiency index ${\text{LPEC}}_{i}^{\left(t\right)}$ as computed from the CognityTrack model. The intervention activation condition is:


(12) $$I_{i}^{\left(t\right)}=\{\begin{array}{ll} 1, & {\text{if LPEC}}_{i}^{\left(t\right)}<\tau \\ 0, & \text{otherwise} \end{array}$$

where τ is a strategic threshold determined via baseline psychological health benchmarking.

The PsyAlign system defines an intervention vector:


(13) $$\begin{array}{l} {\text{v}}_{i}^{\left(t\right)}=\left[\begin{array}{c} T_{i}^{\left(t\right)}\\ C_{i}^{\left(t\right)}\\ R_{i}^{\left(t\right)}\\ F_{i}^{\left(t\right)}\\ M_{i}^{\left(t\right)} \end{array}\right]\in {\left\{0,1\right\}}^{5} \end{array}$$

where each binary flag represents a discrete intervention type:

- *T*: task reconfiguration
- *C*: counseling
- *R*: role realignment
- *F*: feedback calibration
- *M*: managerial empathy reinforcement.

This vector is generated by logical evaluation rules:


(14) $$T_{i}^{\left(t\right)}=\{\begin{array}{ll} 1, & \text{if }s_{i}^{\left(t\right)}>\theta_{1}\wedge f_{i}^{\left(t\right)}<\theta_{2}\\ 0, & \text{otherwise} \end{array}$$

(15) $$C_{i}^{\left(t\right)}=\{\begin{array}{ll} 1, & \text{if }a_{i}^{\left(t\right)}>\theta_{3}\vee \nabla s_{i}^{\left(t\right)}>\theta_{4}\\ 0, & \text{otherwise} \end{array}$$

(16) $$R_{i}^{\left(t\right)}=\{\begin{array}{ll} 1, & \text{if }j_{i}^{\left(t\right)}<\theta_{5}\wedge d_{i}^{\left(t\right)}>\theta_{6}\\ 0, & \text{otherwise} \end{array}$$

(17) $$F_{i}^{\left(t\right)}=\{\begin{array}{ll} 1, & \text{if }\nabla j_{i}^{\left(t\right)}<0\wedge w_{i}^{\left(t\right)}<\theta_{7}\\ 0, & \text{otherwise} \end{array}$$

(18) $$M_{i}^{\left(t\right)}=\{\begin{array}{ll} 1, & \text{if var}(p_{i}^{\left(t-T:t\right)})>\theta_{8}\\ 0, & \text{otherwise} \end{array}$$

The combined organizational intervention matrix at time *t* is then:


(19) $$\begin{array}{l} {\text{V}}^{\left(t\right)}=\left[\begin{array}{c} {\text{v}}_{1}^{\left(t\right)}\\ \vdots \\ {\text{v}}_{n}^{\left(t\right)} \end{array}\right]\in {\left\{0,1\right\}}^{n\times 5} \end{array}$$

Each row vector is dispatched to the HRM system, which maps it to specific operational modules. The intervention strength $\sigma_{i}^{\left(t\right)}$ is defined as the Hamming weight of ${\text{v}}_{i}^{\left(t\right)}$:


(20) $$\begin{array}{l} \sigma_{i}^{\left(t\right)}=\overset{5}{\underset{k=1}{\sum }}v_{ik}^{\left(t\right)} \end{array}$$

To monitor effectiveness, PsyAlign computes a psychological recovery index (PRI):


(21) $$\begin{array}{l} {\text{PRI}}_{i}^{\left(t+\Delta \right)}=\frac{{\text{MWI}}_{i}^{\left(t+\Delta \right)}-{\text{MWI}}_{i}^{\left(t\right)}}{\Delta } \end{array}$$

A positive PRI indicates improvement, and the cumulative intervention success rate (CISR) is defined as:


(22) $$\begin{array}{l} \text{CISR}=\frac{1}{n}\overset{n}{\underset{i=1}{\sum }}{⊮}_{\left[{\text{PRI}}_{i}^{\left(t+\Delta \right)}>0\right]} \end{array}$$

For continuous optimization, the intervention cost-efficiency ratio (ICER) is defined by:


(23) $$\begin{array}{l} {\text{ICER}}_{i}=\frac{{\text{PRI}}_{i}^{\left(t+\Delta \right)}}{\sigma_{i}^{\left(t\right)}\cdot {\text{Cost}}_{i}} \end{array}$$

where Cost_*i*_ denotes the composite resource input to all activated components in ${\text{v}}_{i}^{\left(t\right)}$.

In practical deployment, PsyAlign was integrated into HR operations over two quarters. Psychological tracking tables (as shown in Table 1) were automatically interpreted to assign real-time interventions, supported by AI-driven dashboards.

## 3 Results

### 3.1 Relationship between psychological states and workforce performance

**Figure 1:**
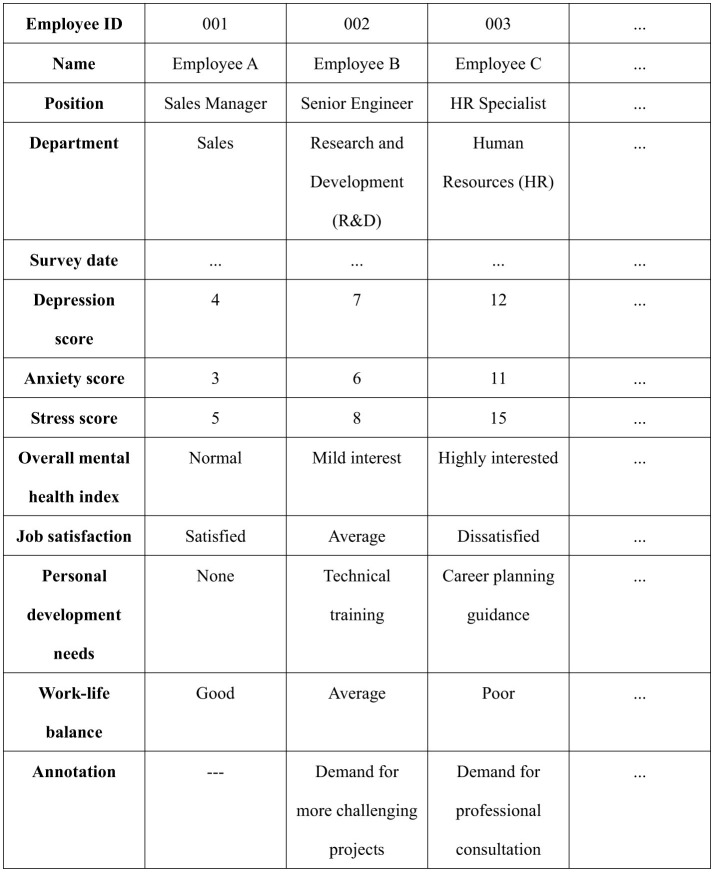
An enterprise's employees' psychological state change tracking record table.

The analysis revealed a strong correlation between employees' psychological states and their levels of work engagement and output (Figure 1). Three principal psychological dimensions were examined in detail: flow, anxiety, and stress. When employees entered a state of flow, characterized by deep immersion, intrinsic motivation, and alignment between skills and task demands, they consistently demonstrated higher levels of concentration and creativity. These individuals often exhibited greater persistence, willingly invested additional time and energy, and delivered work of superior quality. The flow state was positively associated with increased initiative, collaborative behavior, and innovation, all of which significantly enhanced workforce effectiveness. Conversely, employees who experienced cognitive anxiety were more likely to display reduced engagement, difficulty focusing, and impaired decision-making. Anxiety was typically accompanied by heightened self-doubt, fear of failure, and over-attention to potential negative outcomes. As a result, these individuals exhibited erratic work behaviors, frequent task interruptions, and lower productivity levels. Moreover, cognitive anxiety diminished employees' creative problem-solving capacity and limited their ability to handle complex responsibilities effectively. Stress levels produced a more nuanced set of results. Moderate stress appeared to have a mildly positive effect by stimulating competitiveness and urgency, especially when paired with effective coping mechanisms and a supportive environment. However, when stress levels exceeded an optimal threshold, employees reported frequent mental fatigue, emotional volatility, and physical discomfort, which in turn led to increased error rates, poor time management, and deteriorated interpersonal relations. The impact of stress on workforce outcomes thus depended heavily on contextual factors, such as task load, role clarity, and availability of support resources.

### 3.2 Effects of psychological states on work efficiency

Beyond general performance, psychological states also showed marked influence on specific aspects of employee work efficiency. Employees in a flow state exhibited seamless task execution and strong time-on-task ratios. They were capable of avoiding distractions, maintaining steady momentum, and meeting or exceeding deadlines. Their ability to generate novel ideas and adapt strategies during problem-solving led to both higher quality outputs and optimized processes. In contrast, employees experiencing cognitive anxiety frequently encountered concentration lapses and cognitive overload. Their work processes became fragmented due to intrusive thoughts and internal distractions, and they often demonstrated disorganized workflow patterns. Anxiety also affected their judgment, leading to overly cautious or delayed decision-making, and an aversion to taking calculated risks. Consequently, anxiety contributed to inefficiencies in both task execution and communication flow. The impact of stress was similarly disruptive at high levels. Under excessive stress, employees tended to rush assignments without adequate review, which increased the probability of errors and overlooked details. Stress also impaired cognitive functions such as memory, problem-solving, and emotional regulation, further slowing down reaction times and hindering teamwork. In high-pressure settings, prolonged stress led to burnout symptoms and absenteeism, which collectively reduced overall workforce efficiency.

### 3.3 Patterns across psychological states

Collectively, the findings suggest that psychological wellbeing is a critical determinant of individual and organizational performance. Employees who maintained positive psychological states, particularly those in a sustained flow state, were significantly more likely to demonstrate consistent efficiency, creativity, and collaborative behavior. In contrast, those who reported higher levels of anxiety or unregulated stress experienced declines in both qualitative and quantitative performance metrics. These patterns underscore the necessity for organizations to adopt proactive psychological monitoring and support mechanisms. Strategies aimed at fostering flow—such as meaningful goal setting, autonomous task design, and recognition systems—were observed to be effective in elevating work efficiency. Conversely, environments that failed to address anxiety and stress factors faced chronic productivity loss and disengagement risks among their workforce.

### 3.4 Statistical analysis of psychological indicators and work outcomes

To evaluate the strength and significance of the relationships between psychological states and employee work outcomes, we conducted a series of statistical tests using SPSS 26.0. Pearson correlation analysis revealed significant relationships between flow and work efficiency (*r* = 0.63, *p* < 0.01), and negative correlations between anxiety and efficiency (*r* = −0.58, *p* < 0.01). Stress was moderately negatively correlated with efficiency (*r* = −0.44, *p* < 0.05). In addition, job satisfaction was positively correlated with flow (*r* = 0.47, *p* < 0.01) and negatively correlated with anxiety (*r* = −0.53, *p* < 0.01). We then ran a multiple linear regression model with work efficiency as the dependent variable, and flow, anxiety, and stress as predictors. The overall regression model was statistically significant [*F*_(3, 64)_ = 18.56, *p* < 0.001], with an adjusted *R*^2^ of 0.62. Flow had the strongest positive effect on work efficiency (β = 0.51, *p* < 0.001), while anxiety (β = −0.34, *p* = 0.003) and stress (β = −0.21, *p* = 0.046) were both significant negative predictors. Effect sizes were also calculated using Cohen's *f*^2^. The impact of flow on work efficiency yielded a large effect size (*f*^2^ = 0.38), while anxiety and stress showed moderate effect sizes (*f*^2^ = 0.15 and *f*^2^ = 0.08, respectively).

## 4 Discussion

**Figure 2:**
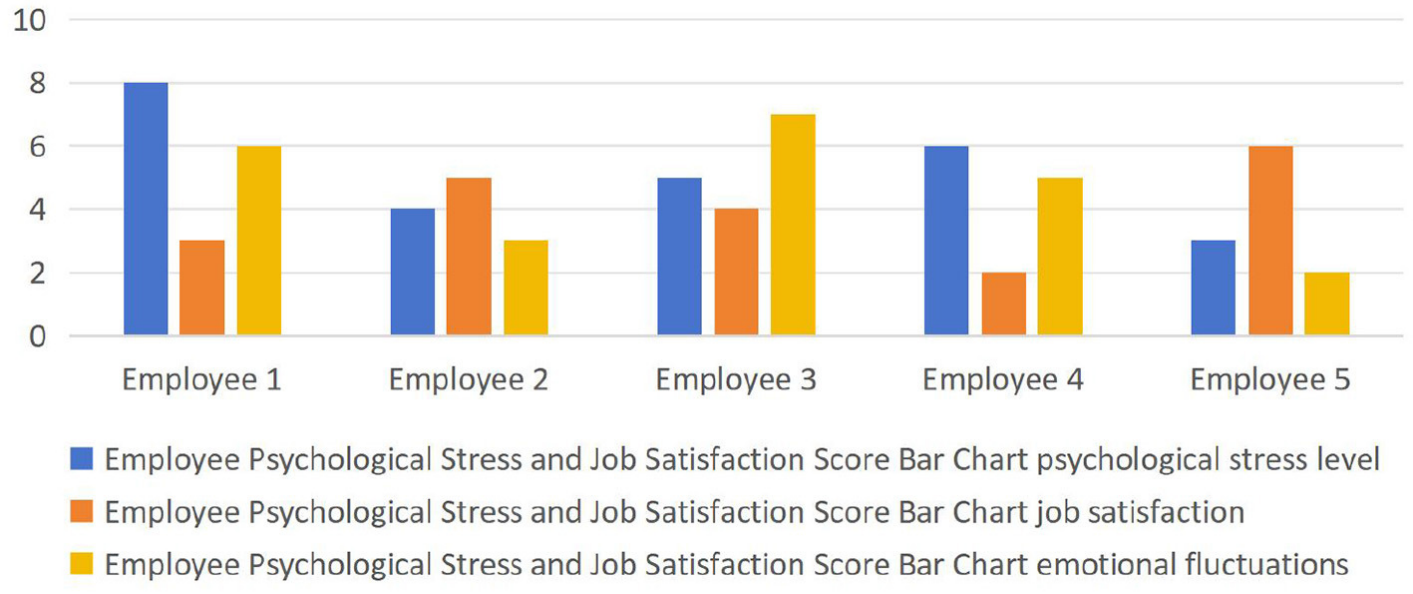
Histogram of the three dimensions of psychological state (stress level, job satisfaction, mood swings) ratings of the employees of an enterprise.

To contextualize our theoretical and empirical findings, we conducted a case analysis in a mid-sized Chinese enterprise. Five employees from different departments were assessed on stress, job satisfaction, and emotional stability, each rated on a 1–10 scale. The resulting histogram (Figure 2) illustrates variation in psychological states across individuals.

Employee 1 exhibited high stress (8), low satisfaction (3), and elevated mood fluctuations (6), suggesting a high-risk profile linked to performance decline and interpersonal strain. This case calls for interventions such as workload adjustment, counseling, and career development planning. Employee 2 showed low stress (4), moderate satisfaction (5), and stable emotions (3), indicating a balanced state requiring no immediate intervention, though continued monitoring is advised. Employee 3 had moderate stress (5), low satisfaction (4), and high emotional fluctuation (7). Emotional instability and dissatisfaction suggest the need for emotional regulation training and coaching support. Employee 4 reported moderately high stress (6), very low satisfaction (2), and moderate mood swings (5), reflecting a mismatch between role and internal motivation. Role revision, feedback, and structured rewards are appropriate responses. Employee 5 showed a healthy profile: low stress (3), high satisfaction (6), and strong emotional stability (2). According to the flow framework, this employee may benefit from more cognitively demanding or leadership tasks.

This case supports our broader findings that psychological indicators meaningfully correlate with observed work behaviors. It reinforces that psychological profiling should inform individualized HR strategies, moving beyond one-size-fits-all models toward adaptive, data-driven management. Our study offers empirical and theoretical evidence that psychological states—particularly flow, anxiety, and stress—are closely linked to employee productivity and efficiency. Through multidimensional assessment and intervention, we demonstrate how psychological diagnostics can support strategic human resource management (SHRM), especially in public health and knowledge-intensive sectors in China. Flow emerged as a key driver of focus, creativity, and persistence. Employees experiencing flow showed higher engagement and satisfaction, aligning with Csikszentmihalyi's theory of optimal experience. Practically, this requires aligning task difficulty with skill, offering timely feedback, and supporting autonomy through job design and leadership that limits unnecessary interruptions. Conversely, anxiety significantly impaired performance by reducing concentration, decision-making, and creativity. Consistent with cognitive overload models, prolonged anxiety leads to emotional exhaustion and poor judgment. Managers must interpret anxiety as an organizational signal, addressing systemic causes such as excessive workload or unclear expectations. Interventions like mindfulness, coaching, and workload reviews should be integrated into HR practices. Stress exhibited a more complex role. While moderate stress could enhance performance under resilience, chronic stress reduced nearly all key metrics. This supports the Yerkes–Dodson law, highlighting an inverted-U relationship between stress and performance. Personalized strategies are essential—resilient individuals may benefit from challenge, while others require simplification or emotional support. Leadership that fosters psychological safety and open communication is critical in managing stress effectively.

Our findings reinforce the notion that strategic HRM is most effective when integrated with psychological diagnostics. Gile et al. (2022a), who found that performance in Ethiopian public hospitals improved when human resources strategies were aligned with employee perceptions and needs, this study further validates that such alignment transcends geography and sectoral boundaries. In the Chinese context, where hierarchical culture and performance pressure are often intensified, applying psychological insights in HRM is both necessary and transformative. Tailored interventions like emotional profiling, personalized incentive plans, and development pathways significantly contribute to motivation, retention, and organizational loyalty. Compared with international studies, several domestic investigations have also highlighted the psychological determinants of workforce performance in Chinese institutions. Zhang and Li (2019) examined psychological stress among Chinese public sector employees and found that high emotional exhaustion was significantly associated with absenteeism and turnover intentions. However, their analysis focused mainly on static cross-sectional data without embedded intervention mechanisms. In contrast, our study extends this line of inquiry by adopting a dynamic psychological tracking model and integrating stress, flow, and anxiety as multidimensional predictors linked to real-time organizational outcomes. This enhances the practical utility of our framework, particularly in high-pressure Chinese organizational environments.

Importantly, this study also emphasizes the systemic value of psychological data in organizational decision-making. Incorporating psychological indicators into workflow design, team composition, and strategic planning allows managers to predict behavioral responses, allocate risk, and align talent with task complexity. This practice contributes not only to operational efficiency but also to employee wellbeing—creating a virtuous cycle where psychological support drives performance, and success reinforces mental health. Another key insight is the interplay between individual and environmental factors. Psychological states are not static traits but dynamic conditions that fluctuate based on context. Thus, continuous assessment—rather than one-time diagnosis—is essential. Organizations should implement dynamic tracking systems to monitor changes in stress, anxiety, or satisfaction, and respond with adaptive HR measures. Digital tools such as AI-driven wellbeing platforms and emotion-sensitive feedback systems can enhance responsiveness and personalization in this domain.

The study also sheds light on the importance of leadership behavior. Managers who adopt emotionally intelligent leadership styles—such as coaching, empathetic listening, and non-punitive feedback—are better positioned to foster psychologically resilient teams. Moreover, training mid-level managers to interpret psychological data and implement localized interventions may bridge the gap between HR strategy and day-to-day team dynamics. From a broader theoretical standpoint, this research supports a biopsychosocial perspective of workforce performance. It illustrates that organizational efficiency is not only an economic or structural phenomenon, but also a psychological and social construct. The application of psychological state analysis—when embedded within HRM systems—can move organizations toward a more holistic, employee-centered model of performance enhancement. Despite these contributions, the study is subject to several limitations. The primary limitation lies in the scope and scale of data, which was restricted to a single enterprise in a specific cultural context. The psychological assessments, though multidimensional, relied on self-report data, which may introduce bias due to social desirability or response fatigue. Future research should incorporate longitudinal and cross-sectoral studies to examine the temporal dynamics of psychological states and their broader applicability. Experimental studies could also assess the causal efficacy of different interventions, such as the comparative impact of individual coaching vs. structural workflow changes. This study demonstrates the strategic value of integrating psychological state analysis into organizational management. By recognizing the cognitive and emotional dimensions of employee experience, organizations can design interventions that enhance productivity while promoting health and satisfaction. As work continues to evolve in complexity and uncertainty, placing psychological wellbeing at the center of HRM will be key to sustaining resilient, adaptive, and high-performing organizations.

In the healthcare sector, where psychological resilience is frequently tested due to life-critical responsibilities and high patient volumes, integrating mental health diagnostics into HRM is especially relevant. For instance, hospital HR departments can implement regular stress screenings, establish psychological support teams, and use real-time workload data to adjust nurse rotations. The findings from this study offer a transferrable framework to such environments, supporting the argument that psychologically informed HRM strategies can enhance not only staff retention and wellbeing, but also the quality of patient care.

Compared to prior research conducted in Chinese universities and public hospitals (e.g., Zhang and Li, 2019; Liu et al., 2021), our study presents a more integrative approach by linking individual psychological metrics to organizational strategy execution. While previous studies have focused on isolated dimensions such as stress or burnout, our multi-indicator model allows for simultaneous tracking and targeted interventions, offering a more practical solution for HR managers and policymakers.

The statistical results offer quantitative support for our theoretical framework. As shown in the regression analysis, flow was the strongest predictor of work efficiency, with a large effect size and a significant positive standardized coefficient (β = 0.51, *p* < 0.001). This finding confirms that when employees are immersed in their tasks and experience intrinsic motivation, their productivity improves markedly. Conversely, both anxiety and stress were negatively associated with work outcomes, though anxiety showed a slightly stronger effect (β = −0.34, *p* = 0.003) than stress (β = −0.21, *p* = 0.046). These results align with prior studies linking cognitive overload and emotional tension to decreased task performance and decision-making capacity. Furthermore, the correlation analysis indicated that job satisfaction was positively associated with flow and negatively associated with anxiety, suggesting that psychological states also mediate affective work outcomes. Collectively, these findings empirically validate the hypothesized relationships and provide robust evidence for integrating psychological diagnostics into workforce management strategies.

While our findings indicate that anxiety and stress are generally associated with lower work efficiency, it is important to acknowledge that these effects may vary across individuals. Prior research suggests that personality traits—such as resilience, conscientiousness, and emotional stability—can moderate how stress influences performance outcomes. For example, employees with high self-regulation capacity may perform well under pressure, using stress as a motivator to achieve goals. Others may possess strong coping mechanisms, such as problem-focused or emotion-focused strategies, which buffer the negative impact of psychological strain. Leadership style is another critical moderating factor. Transformational leadership, characterized by individualized consideration and motivational support, has been shown to reduce stress responses and enhance engagement. Managers who foster psychological safety and autonomy may enable employees to interpret high-demand situations as challenges rather than threats. Future research could investigate these moderating variables through multi-level modeling or interaction term analysis, to develop more personalized intervention strategies within organizations.

### 4.1 Practical implications and actionable recommendations

Based on the findings, we propose several concrete strategies for organizations aiming to monitor and improve employee psychological states. Organizations should adopt structured psychological tracking mechanisms. This could involve quarterly digital assessments using validated tools like the DASS-21, integrated into internal HR platforms or employee self-service systems. Scores can be anonymized and aggregated at the departmental level to identify patterns of stress or disengagement. HR managers can implement early warning systems that trigger automated alerts when an employee's psychological score declines significantly across time points. These alerts could prompt check-ins, mental health consultations, or workload redistribution. Goal-setting processes should incorporate employee input and psychological readiness. For instance, weekly task plans can be accompanied by brief self-reflections on energy levels and motivation, allowing supervisors to adjust task assignments dynamically. Team leaders should receive training in psychologically intelligent leadership, including recognizing signs of emotional fatigue, providing non-punitive feedback, and facilitating open discussion spaces during team meetings. Companies can introduce low-cost wellbeing interventions, such as mindfulness sessions, resilience workshops, or access to external counseling hotlines. These initiatives not only enhance wellbeing but signal institutional commitment to mental health. By embedding these practices into regular workflows and performance management systems, organizations can move from reactive to preventive psychological support, creating a more adaptive, motivated, and resilient workforce.

### 4.2 Limitations and future directions

This study is subject to several limitations that should be acknowledged. The data were collected from a single mid-sized enterprise in Eastern China, which may limit the generalizability of the findings to other organizational settings, industries, or cultural contexts. Future studies could broaden the scope by including participants from multiple sectors and geographic regions to enhance external validity. The use of self-reported surveys introduces potential response bias, including social desirability effects and common method variance. While anonymity was ensured, future research may benefit from incorporating multi-source data, such as supervisor ratings or behavioral performance metrics. Although our research included repeated psychological tracking over time, the study was not experimental in design. As a result, causal inferences about the directionality of relationships among psychological states and work outcomes should be drawn cautiously. Future studies employing longitudinal or experimental designs could more definitively establish causality. This study did not measure potential moderating variables such as personality traits, coping mechanisms, or leadership styles, which could influence how psychological states affect work performance. Including these variables in future models could lead to more personalized and precise intervention strategies.

## 5 Conclusion

This study underscores the significant role of psychological state analysis in organizational management, offering both theoretical insights and empirical validation. Through a multidimensional evaluation of employees' psychological conditions—namely flow, anxiety, and stress—we identified a direct and measurable link between mental wellbeing and work performance. Positive psychological states such as high engagement, satisfaction, and emotional stability consistently contributed to higher work efficiency, while unmanaged stress and anxiety impeded both cognitive function and task execution. These findings reinforce existing behavioral models and expand them into a strategic HRM framework. The case-based illustration further substantiated that tailored psychological profiling and responsive interventions lead to meaningful improvements in employee behavior and organizational productivity. By advancing a biopsychosocial perspective, this study contributes to the growing recognition that psychological health is not a peripheral concern but a foundational component of sustainable performance. From a practical standpoint, the integration of psychological state monitoring into human resource strategies provides a clear roadmap for enhancing workforce engagement and efficiency. Organizations are advised to adopt a holistic approach that combines regular psychological assessments, employee development planning, stress management programs, and emotionally intelligent leadership practices. For managers and policymakers—especially within high-pressure sectors like healthcare, education, and public administration—these findings advocate for the institutionalization of mental health support within operational models. Creating flexible work systems, offering personalized incentive structures, and embedding emotional feedback mechanisms are not just beneficial, but essential for building resilient, future-ready organizations. Moreover, policies at the organizational and governmental levels should encourage data-driven wellbeing frameworks that treat psychological safety and motivation as core dimensions of performance management. As the demands on human capital continue to evolve, organizations that prioritize psychological insight will be better equipped to navigate complexity, maintain competitive advantage, and foster enduring employee commitment.

To translate these findings into actionable strategies, we recommend that organizations—especially in high-pressure sectors such as healthcare, education, and public service—develop tailored psychological assessment protocols and embed them into routine HRM systems. Managers should be equipped with tools and training to interpret psychological indicators and respond with personalized interventions. Furthermore, digital wellbeing platforms and AI-based feedback systems could be explored to streamline psychological tracking and resource allocation in real time. Future research should expand this model by incorporating larger, more diverse samples and exploring longitudinal effects of psychological interventions on organizational performance. Comparative studies across industries and national contexts would also enhance generalizability. In addition, further investigation into the use of technology, such as emotion-sensitive systems and real-time stress analytics, would provide valuable insight into how psychological diagnostics can be scaled and adapted for dynamic work environments. This study serves as a foundation for a growing research agenda that places mental wellbeing at the core of strategic human resource development.

## Data Availability

The original contributions presented in the study are included in the article/supplementary material, further inquiries can be directed to the corresponding author.
